# Early Hospital Discharge on Day Two Post Robotic Lobectomy with Telehealth Home Monitoring: A Pilot Study

**DOI:** 10.3390/cancers15041146

**Published:** 2023-02-10

**Authors:** Edoardo Bottoni, Giuseppe Mangiameli, Alberto Testori, Federico Piccioni, Veronica Maria Giudici, Emanuele Voulaz, Nadia Ruggieri, Francesca Dalla Corte, Alessandro Crepaldi, Giulia Goretti, Elena Vanni, Martina Pisarra, Umberto Cariboni, Marco Alloisio, Maurizio Cecconi

**Affiliations:** 1 Division of Thoracic Surgery, IRCCS Humanitas Research Hospital, Via Manzoni 56, Rozzano, 20089 Milan, Italy; 2 Department of Biomedical Sciences, Humanitas University, Via Rita Levi Montalcini 4, Pieve Emanuele, 20090 Milan, Italy; 3 Anesthesia Unit 1, Department of Anesthesia and Intensive Care, IRCCS Humanitas Research Hospital, Via Manzoni 56, Rozzano, 20089 Milan, Italy; 4 Quality Department, IRCCS Humanitas Research Hospital, Via Manzoni 56, Rozzano, 20089 Milan, Italy; 5 Humanitas Clinical and Research Center-IRCCS, Via Manzoni 56, Rozzano, 20089 Milan, Italy; 6 Department of Economics, Management and Quantitative Methods, University of Milan, Via Conservatorio 7, 20122 Milan, Italy

**Keywords:** early discharge, tele monitoring, telemedicine, telehealth home monitoring thoracic surgery, oncological surgery, lung cancer, NSCLC

## Abstract

**Simple Summary:**

Actually, the reported postoperative length of stay after robotic lobectomy is 4 days even in highly specialized centers. Several innovations have been recently introduced in the field of surgery to improve surgical outcome, between these the more remarkable is certainly the adoption of telemedicine. During the Coronavirus pandemic several wearable sensors and mobile applications were available to monitor physical and psychological parameters at home or in hospital. Despite this, perioperative telemonitoring is still rarely used in clinical practice, especially in thoracic surgery. Herein, we report the preliminary feasibility results of a pilot study for a protocol of early discharge (on day 2) with telehealth home monitoring after robotic lobectomy for cancer. During the study period, 10 patients satisfied all preoperative clinical and postoperative discharge criteria. No postoperative complication occurred neither readmission. Our preliminary results confirm as the integration of telehealth home monitoring in a fast-track protocol allows a safe discharge on postoperative day 2 after robotic surgery for cancer. The selection of patients is crucial for the success of this approach which remains applicable to VATS surgery too. A potential economic benefit for the health system could derive from this protocol if this data is confirmed in a larger sample.

**Abstract:**

Despite the adoption of enhanced recovery programs, the reported postoperative length of stay after robotic surgery is 4 days even in highly specialized centers. We report preliminary results of a pilot study for a new protocol of early discharge (on day 2) with telehealth home monitoring after robotic lobectomy for lung cancer. All patients with a caregiver were discharged on postoperative day 2 with a telemonitoring device if they satisfied specific discharge criteria. Teleconsultations were scheduled once in the afternoon of post-operative day 2, twice on postoperative day 3, and then once a day until the chest tube removal. Post-discharge vital signs were recorded by patients at least four times daily through the device and were available for consultation by two surgeons through phone application. In case of sudden variation of vital signs or occurrence of adverse events, a direct telephone line was available for patients as well as a protected re-hospitalization path. Primary outcome was the safety evaluated by the occurrence of post-discharge complications and readmissions. Secondary outcome was the evaluation of resources optimization (hospitalization days) maintaining the standard of care. During the study period, twelve patients satisfied all preoperative clinical criteria to be enrolled in our protocol. Two of twelve enrolled patients were successively excluded because they did not satisfy discharge criteria on postoperative day 2. During telehealth home monitoring a total of 27/427 vital-sign measurements violated the threshold in seven patients. Among the threshold violations, only 1 out of 27 was a critical violation and was managed at home. No postoperative complication occurred neither readmission was needed. A mean number of three hospitalization days was avoided and an estimated economic benefit of about EUR 500 for a single patient was obtained if compared with patients submitted to VATS lobectomy in the same period. These preliminary results confirm that adoption of telemonitoring allows, in selected patients, a safe discharge on postoperative day 2 after robotic surgery for early-stage NSCLC. A potential economic benefit could derive from this protocol if this data will be confirmed in larger sample.

## 1. Introduction

Mini-invasive surgery has become standard of care for pulmonary lobectomy as consequence of the clear advantages that have been demonstrated in recent randomized clinical trials [1]. During the last two decades, mini-invasive surgical techniques and protocols for enhanced recovery after thoracic surgery (ERAS) have been combined with the aim of decreasing the length of hospital stay (LOS), complication and readmission rates [2]. Despite these innovations, the reported postoperative length of stay after robotic lung lobectomy is about 4 days even in highly specialized centers [3,4,5]. Experience of early discharge (lower than 4 days) after robotic surgery are lacking, whereas after VATS lobectomy for lung cancer, a discharge on postoperative day 2 has been reported in 46% of patients in a Danish study [6]. The medical factors (bleeding, arrythmia, oxygen dependency and uncontrolled pain) that commonly prevent an early discharge are commonly reported in the first 2 days of stay. Several non-medical factors also prevent an early discharge and his related advantages. Among these the most common reported are cultural (patient and family fears, lack of extra hospital care organization), economic (no incentive for the hospital to shorten LOS) and geographic (distance of care structures, remoteness of the home, poor availability of appropriate structures for convalescence). 

Conversely, several innovations have been recently introduced in the field of surgery to improve surgical outcome, between these the more remarkable is certainly the adoption of telemedicine [7]. During the Coronavirus 2019 (COVID-19) pandemic period several wearable sensors and (mobile) applications were available to monitor physical and psychological parameters at home or in hospital [8]. Despite this, perioperative telemonitoring is currently still rarely used in clinical practice, especially in thoracic surgery [9].

Herein, we report the preliminary feasibility results of a pilot study for a protocol of early discharge with telehealth home monitoring after robotic lobectomy for cancer. 

## 2. Methods

### 2.1. Study Design and Patients

In this single-center quality improvement pilot study, all patients underwent robotic lobectomy for lung cancer at the division of Thoracic Surgery of IRCCS Humanitas Research Hospital and were discharged in postoperative day 2 with telehealth home monitoring (ADITECH/ADiLife device). A postoperative day 2 was chosen because of after revision of our robotic lobectomy series. We have identified postoperative day 2 as threshold value for discharge capable of excluding the majority of early postoperative complication. In our series, they were bleeding, postoperative arrythmia (atrial fibrillation), oxygen dependency and uncontrolled pain. The aim is to include 50 patients during a period of one year of surgical activity. A preliminary analysis of data was scheduled after the inclusion of 10 patients, whereas it was established that the study would be suspended in event of a 20% readmission rate. The protocol was approved by the Ethical Committee of the IRCCS Humanitas Research Hospital (research register number #201900432), and this study was conducted in accordance with the SQUIRE guidelines and the Declaration of Helsinki [10]. All patients signed an informed consent. Patient characteristics were collected at the face-to-face baseline assessments. Clinical and surgical data were collected from medical records, including in-hospital and post-discharge complications within 30 days after surgery, the hospital readmission within 30 days after surgery and timing of post-discharge complications and hospital readmissions [11].

### 2.2. Inclusion and Exclusion Criteria

Eligible patients satisfied the following criteria: a performance status ECOG 0–2, age between 18 and 75 years, confirmed histopathological diagnosis of NSCLC with a clinical T1-2N0M0 staging, scheduled for lobectomy, availability of internet access, of a smartphone and of a caregiver living with the patient during the study, both at less than 60 Km from the hospital. All patients presenting the above-mentioned inclusion criteria were enrolled on this protocol and submitted to robotic lobectomy, according to our technique [12].

On postoperative day 2, the enrolled patients were discharged with a telemonitoring device if they satisfied the follow criteria: pain control (Numerical Rating Scale < 7), social context with the availability of a caregiver, systolic blood pressure > 95 or <160 mmHG, temperature < 37 °C, heart rate < 100 bpm and peripheral oxygen saturation > 92%. Exclusion criteria were cancellation of scheduled surgery, intraoperatory conversion to open surgery due to adherences or major complication (bleeding > 2000 mL, anesthetic complications needing reintubation after surgery or surveillance in the intensive care unit), the occurrence of postoperative complication preventing a safe discharge and perceived incapability to use components of the remote home monitoring system due to visual or cognitive impairment.

### 2.3. Protocol

A specific enhanced recovery protocol was adopted based on specific items covering topics related to pre-admission, admission, intraoperative care and postoperative care. Pre-operative surgical and anesthetic consultations were systematically performed to offer a pre-operative counselling to diminish fear, fatigue, and pain. A QR code was provided to patients to download multimedia information containing explanations of respiratory physiotherapy’s procedures which were started at least one week before surgery.

All surgical procedures were performed under general anesthesia adopting lung-protective ventilation (tidal volume 4–6 mL/kg predicted body weight, positive end-expiratory pressure—PEEP between 5 and 8 cmH_2_O, fraction of inspired oxygen between 0·5 and 0·8) with a Da Vinci Xi system (Intuitive Surgical, Sunnyvale, CA, USA). Analgesia was assured through a multimodal protocol including: erector spinae plane (ESP) block (levobuvicaine 0·25% 20 mL), dexamethasone 4–8 mg, sulphate magnesium 1 g before surgical incision and ketorolac 30 mg and paracetamol 1 g 30 min before awakening. Non-steroidal anti-inflammatory drugs and paracetamol were continued during post-operative period. At the end of surgery, a 28 Fr chest tube was systematically placed. A Heimlich valve was placed on postoperative day 1 and the patient discharged with chest tube if on X-ray the lung reaches the thoracic wall even in presence of air leak. If daily pleural effusion was less than 200 cc without air leakage, the chest drain was removed before discharge.

### 2.4. Telehealth Home Monitoring

The remote home monitoring consisted of both teleconsultation and analysis of data recorded by a device (ADITECH/ADiLife, Ancona, Italy) [13]. All patients were informed of the need to use a device during the study period and a caregiver was involved in his use. The device was provided to the patient only the day before surgery to avoid emotional stress. All patients and all their caregiver were trained to use the device during hospitalization. The data obtained during familiarization period have been clearly excluded from the analysis.

A first, teleconsultation was performed the day of discharge, after patient’s returning home. On postoperative day 3, a teleconsultation was performed twice, in the morning and in the afternoon. Starting from postoperative day 3, once a day, until the chest tube was removed in outpatients visit. The chest tube was removed if the daily output was lower than 200 cc without air leakage. 

Data were not monitored in real time, post-discharge vital signs (blood pressure, temperature, heart rate, and peripheral oxygen saturation) and patient-reported symptoms were self-recorded by patients at least four times daily through the device. Monitoring data were visible to patients and regularly checked by the case managers (research physician) through dedicated application. An alert signal was directly received through research physician mobile phone (both text and e-mail) in case of abnormality of recorded vital signs (threshold violations). Threshold violations were defined as moderate (yellow) and critical (red). Yellow threshold violations were defined as: systolic blood pressure > 140 or <100 mmHG, diastolic blood pressure > 95 or <60 mmHg; oximetry < 94%; heart rate > 100 bpm and temperature > 37.5 °C. Red threshold violations were defined as: systolic blood pressure >160 or <80 mmHg, diastolic blood pressure >110 or <40 mmHg; oximetry <89%; heart rate > 140 bpm and temperature > 38 °C. In these latter cases, patients were contacted by telephone to obtain additional information regarding parameter deviations. The same procedure was followed if data were missing to provide technical assistance. Furthermore, in case of sudden variation of vital signs or occurrence of adverse events a direct telephone line was available 24 h for patients as well as a protected re-hospitalization path. After the removal of chest drain all patients were followed according to the standard of care. A 30-days follow up was available for all enrolled patients. 

### 2.5. Outcome Measures

Primary outcome measure was the safety of this protocol evaluated by the occurrence of post-discharge complications and number of hospital readmissions. Secondary outcome was the evaluation of resources optimization (hospitalization days and costs saved) maintaining the standard of care.

### 2.6. Statistical Analysis

Descriptive statistics were used to present baseline and surgery characteristics of patients. Continuous variables are reported as mean and standard deviation variables are reported as number and percentage. Cost items were reported in euro. Data analysis was performed with Microsoft Excel software. Statistical significance was assumed for *p* < 0.05. 

## 3. Results

### 3.1. Enrolment and Drop Out

**Figure 1:**
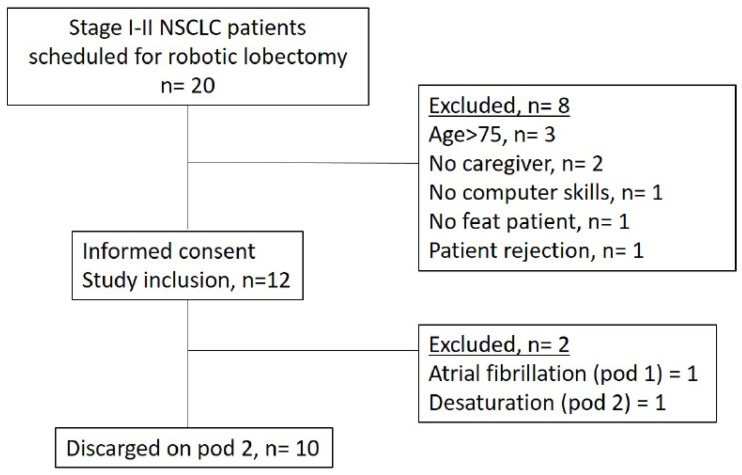
Enrolment and drop out flowchart.

From the beginning of the study (July 2022), 12 of 20 eligible patients consented to participate in the study. The main reasons for ineligibility were age >75 years (*n* = 3), lack of a caregiver (*n* = 2), unfit patient (*n* = 1); whereas the main reasons for refusal were perceived high mental burden (*n* = 1) and insufficient digital skills (*n* = 1). The 12 included patients had a mean age of 66.9 ± 6.3 years with a mean BMI of 26.1 ± 6.3, and 8 (66%) were male. After informed consent was obtained and patients enrolled, two patients dropped out of the study: the first one because of the occurrence on postoperative day 2 of atrial fibrillation, the second one for the occurrence on postoperative day 2 of a severe desaturation. Thus, a total of 10 patients were included in the study and discharged on postoperative day 2. Patients’ clinical and pathological features such as a detailed list of operations performed is presented in Table 1. Figure 1 reports the flowchart with enrolment and drop out. 

### 3.2. Postoperative Period

After surgery, all patients were extubated on the operating table and transferred to a surgical ward after a chest X-ray was received from recovery room. No complications occurred during the first 36 h of hospitalization.

### 3.3. Teleconsultation

The adherence rate to planned teleconsultation was 100%. A total of 41 teleconsultations was performed for a total of 10 patients. In nine patients it was possible to remove chest drain on postoperative day 4 after the four planned teleconsultations. An adjunctive teleconsultation was performed only in one case considering that the drain was removed on post-operative day 5. 

No unplanned teleconsultations were needed. 

### 3.4. Telemonitoring of Vital Signs

**Figure 2:**
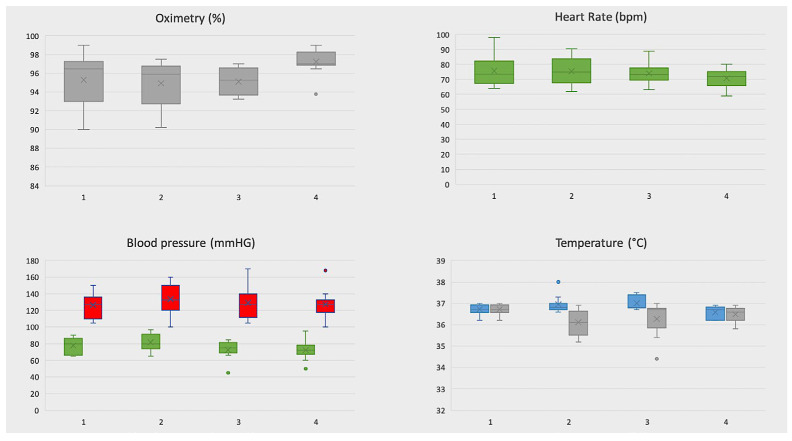
Graphical representation of measurement for each vital sign. The box is bounded by the first (bottom) and third (top) quartiles. The median is represented as a line in the box whereas the mean as a X. In red systolic pression, in green diastolic pression, in blue higher temperature, in gray lower temperature.

The adherence rate to vital signs control was of 100%. Each patient has performed a mean number of 4 measurements per day. A total of 27/427 vital sign measurements violated the threshold in seven patients (Figure 2). Among the threshold violations, only 1 out of 27 was a critical violation. Oximetry threshold violations were observed in 18/101 (17.8%) measurements in six patients. Heart rate threshold violations were recorded in 2/106 (1.8%) measurements in two patients whereas only one blood-pressure threshold violation (1/110; 0.9%) was observed in one patient. Finally, 2/110 (1.8%) threshold violations of temperature were recorded in one patient. In this case, one out of two was a critical violation (Temperature 38 °C). The patient was immediately contacted by phone but the self-measurement of temperature with another device did not confirm this data. 

### 3.5. Complication Rate and Readmission

After discharge, the chest drain was removed on post-operative day 4 for 9 of 10 patients. Only in one case was the chest drain removed on post-operative day 5 due to ongoing (>200 cc/24 h) serum production. During the period of early discharge (from postoperative day 2 to the day of chest tube ablation) no complications were detected neither readmissions were needed. In the period following the removal of the drainage, no complications or readmissions were recorded at 30 days. 

### 3.6. Costs Analysis

The ROBOTIC group was compared with a group of 45 patients submitted to VATS lobectomy for NSCLC (stage I–II without age exclusion criteria) in the study period. The mean cost in euro of each hospitalization per group was estimated. All items considered in the cost analysis are shown in Table 2, they are expressed in mean cost (euros) and as percentage of finally hospitalization costs. The average hospitalization costs per patient was EUR 6,309, which was lower than VATS patients with a mean number of 3 hospitalizations days avoided and an estimated economic benefit of about EUR 500 for single patient. The device cost was of EUR 5,000 for two instruments (*n* = 2) which will be property of thoracic surgery department. 

## 4. Discussion

In the present study we have reported the adoption of telehealth home monitoring applied to a program of early discharge on post-operative day 2 after robotic lobectomy for lung cancer. 

Our study originated from the idea of combining several emerging needs during the COVID-19 pandemic. The first one was the need to optimize the rate of bed occupation to prevent the delay of treatment in oncological cases that was described in Lombardy (Italy) and worldwide during the pandemic [14]. The second one was the needing of early discharge of frail oncological patients to significantly reduce the infectious risk of COVID-19 and non-COVID-19 illnesses related during postoperative period [15]. Last but not least, a further input to design this study was given by the increased use of telemedicine emerged during the pandemic [16].

In this latter scenario, the telehealth home monitoring was identified as a useful tool to reduce a postoperative length of stay that remains as 4 days, even in highly specialized robotic centers [3,4,5]. To date, as well as medical factors, non-medical factors are responsible for a failure of an early discharge. Conversely, we know that the medical factors that commonly prevent an early discharge (atrial fibrillation, bleeding, oxygen dependency and uncontrolled pain) are commonly reported in the first 2 days of stay. For all these reasons, a postoperative on day 2 was chosen as a threshold value for discharge adopting telehealth home monitoring to overcome non-medical limitations and to reduce the length of stay to 2 days.

Several studies have recently described and investigated the utility of telemonitoring during post-surgical period targeting patients undergoing cardiac or orthopedic surgery [17]. With increasing interest, studies focus on perioperative telemonitoring in patients before or, mainly, after abdominal surgery as well [18,19,20,21,22,23,24,25,26]. To the best of our knowledge, our study is the first that has investigated the utility of telemedicine applied to postoperative surveillance in major thoracic surgery. Thus, the main objective of the presented study was to investigate in terms of feasibility and safety our approach. The preliminary results seem to confirm the safety of this approach considering that no adverse events, including complications and readmission, occurred during telemonitoring period. This research aims to enroll 50 patients to assess the security and feasibility of perioperative telemonitoring interventions for thoracic surgery. The goal is to gather evidence on its effectiveness and provide guidance for implementing this technology in healthcare practices. In our opinion, our protocol represents what could become day/overnight surgery for major thoracic surgery performed in the early stages of the disease. The association of robotics, and telehealth home monitoring with automatic transmission of vital parameters, are the “mix technology” which will be of vital importance to implement and improve this path. 

If this study confirms the systematic applicability of this approach, then the derived advantages will be not only for patients but for the health system too. As reported in our cost analysis, there were lower costs for home care with significant economic savings for the healthcare system. The benefit was not only direct (the reduction in hospitalization days) but also indirect because of the optimization of beds for further surgical procedures. If adopted, our protocol will allow a reduction in waste of physical resources in addition to better use of the same (beds, meals, drugs) and a decreased risk of nosocomial infections by reducing the average hospital stay of patients. Furthermore, our protocol, designed specifically for robotic surgery, has the potential to overcome the actual limitations of high costs associated with this technology, thus enhancing its widespread adoption [27]. Obviously, it can also be applied to all minimally invasive techniques to further increase the benefits of mini-invasive approach. 

With the growing adoption of telemedicine, there is an anticipated shift from institutional care towards remote care, as well as an integration of telemedicine with traditional in-person care. Given the ubiquitous spread of the internet in the future, telemedicine will shift care from hospitals and clinics to homes and mobile devices [28].

However, some current difficulties need to be overcome to increase the effectiveness and therefore the use of perioperative telemonitoring in thoracic surgery. 

The major difficulty remains that the main beneficiaries in oncological setting remain who usually the last to adopt innovations such as older with less educational attainment and lower socioeconomic status. In our study, the adoption rate for both teleconsultation and home monitoring system were higher than normal. No issues related to the use of the adopted device were reported by patients. All scheduled teleconsultations were performed, and vital signs were sent more than planned through the device. It showed that the adoption of a telemedicine program was perceived by patients as stimulating but, at the same time, underlines the importance of a caregiver to assure the compliance in the care process. In this scenario, the carefully selection of patients is crucial for the success of this approach considering that perioperative telemonitoring should not be used as a goal itself, but a support for personalized care nowadays. A second limit is a direct consequence of this selection process. Telemonitoring studies often suffer from selection bias towards highly educated patients, leading to reduced external validity and limited generalizability for widespread use [29,30]. Currently, the best target patients for telemonitoring are defined by their ability to use technology and the presence of a caregiver living near the hospital.

Additionally, new feasibility and usability studies are necessary to ensure end-user adoption and integration into workflow, as well as for conducting clinical trials.

Finally, the widespread of telemedicine should require investment in money and time to develop strategies for the implementation and adoption of telemonitoring both in oncological centers and in the surgical community.

## 5. Conclusions

Our preliminary results confirm as the integration of telehealth home monitoring in a fast-track protocol allows a safe discharge on postoperative day 2 after mini-invasive robotic surgery for early-stage NSCLC. The selection of patients is crucial for the success of this approach which remains applicable to VATS surgery too. A potential economic benefit for the health system could derive from this protocol if this data is confirmed in a larger sample.

## Figures and Tables

**Table 1:** Patients’ clinical and pathological features.

Patient	Age	Sex	ASA	BMI	Smoke	cTNM	pTNM	Histology	Surgery	Surgical Time	Chest Tube Removal (POD)	Complication
1	62	M	III	23	Active	T1N0	T1cN0	Spino	RUL	139	IV	0
2	70	M	III	24	Former	T1N0	T1bN0	ADK	RUL	169	IV	0
3	74	F	IV	14	Active	T1N0	T1bN0	ADK	RUL	101	IV	0
4	73	F	III	27	Former	T2N0	T2aN0	ADK	RLL	115	IV	0
5	67	M	III	38	Former	T2N0	T2aN0	ADK	RLL	166	V	0
6	71	M	I	22	Former	T2N0	T2aN0	ADK	RUL	118	IV	0
7	70	F	I	29	No	T1N0	T1bN0	ADK	RUL	157	IV	0
8	65	M	III	33	Active	T2N0	T1aN0	ADK	RUL	111	VI	AF (POD II) Desaturation (POD II-III)
9	58	M	I	26	No	T1N0	T1bN0	Typical Carcinoid	LUL	252	II	0
10	69	M	I	30	Former	T1N0	T2aN0	ADK	RUL	245	VII	Desaturation (POD II)
11	71	F	I	22	Former	T1N0	T1bN0	ADK	RUL	90	IV	0
12	53	M	III	26	Active	T1N0	T1bN0	ADK	LUL	170	IV	0

**Table 2:** Cost items.

Items	Robotic Group (*n* = 10)	VATS Group (*n* = 45)
Average stay (days)	3.00	7·69
Blood products (*n*°)	0 (0%)	25 ± 134 (0.3%)
Drugs (including VAT)	309 ± 11 (4.9%)	361 ± 94 (5.3%)
Medical device (including VAT)	2878 ± 0 (45.6%)	1647 ± 0 (24.4%)
Costs related to medical assistance (euros)	560 ± 45 (8.9%)	855 ± 235 (12.7%)
Hospitalization costs (days of stay)	528 ± 0 (8.4%)	1416 ± 768 (21.0%)
Diagnostic exams	859 ± 235 (13.6%)	1168 ± 609 (17.3%)
Operating room	1174 ± 156 (18.6%)	1285 ± 150 (19.0%)
Average costs	6309 (100.0%)	6756 (100.0%)

Average costs are expressed in euros.

## Data Availability

Derived data supporting the findings of this study are available from the corresponding author [G.M] on request.
